# A Review of Isothermal Amplification Methods and Food-Origin Inhibitors against Detecting Food-Borne Pathogens

**DOI:** 10.3390/foods11030322

**Published:** 2022-01-24

**Authors:** Ye-Ji Moon, So-Young Lee, Se-Wook Oh

**Affiliations:** Department of Food and Nutrition, Kookmin University, Seoul 136-702, Korea; yjm0316@kookmin.ac.kr (Y.-J.M.); leesoyoung0423@kookmin.ac.kr (S.-Y.L.)

**Keywords:** molecular amplification, detection method, food metrix, bacteria, inhibition

## Abstract

The isothermal amplification method, a molecular-based diagnostic technology, such as loop-mediated isothermal amplification (LAMP) and recombinase polymerase amplification (RPA), is widely used as an alternative to the time-consuming and labor-intensive culture-based detection method. However, food matrices or other compounds can inhibit molecular-based diagnostic technologies, causing reduced detection efficiencies, and false-negative results. These inhibitors originating from food are polysaccharides and polyphenolic compounds in berries, seafood, and vegetables. Additionally, magnesium ions needed for amplification reactions can also inhibit molecular-based diagnostics. The successful removal of inhibitors originating from food and molecular amplification reaction is therefore proposed to enhance the efficiency of molecular-based diagnostics and allow accurate detection of food-borne pathogens. Among molecular-based diagnostics, PCR inhibitors have been reported. Nevertheless, reports on the mechanism and removal of isothermal amplification method inhibitors are insufficient. Therefore, this review describes inhibitors originating from food and some compounds inhibiting the detection of food-borne pathogens during isothermal amplification.

## 1. Introduction

Food-borne pathogens, such as *Salmonella* spp., *Escherichia coli* O157:H7, and *Listeria monocytogenes,* threaten public health, causing different food-borne diseases and deaths [1]. Since small amounts of bacteria are found in food, detection methods capable of detecting even small bacterial quantities are needed [2]. The culture-based detection method, a gold standard for pathogen detection, is time-consuming and labor-intensive [3]. Therefore, polymerase chain reaction (PCR), which was developed as a rapid detection technology based on nucleic-acid bond alignment, greatly reduces detection time, and improves the sensitivity and specificity to increase the detection efficiency of food-borne pathogens [4,5]. PCR amplifies nucleic acids through denaturation, annealing, and expansion steps during different temperature cycles, but different temperature cycles can interfere with the nucleic acid amplification process [6,7,8].

An isothermal amplification method was developed to be conducted at a single temperature, which was subsequently improved by replacing the PCR thermocycling step with *Bacillus stearothermophylus* (*Bst*) DNA polymerase, phi DNA polymerase, helicase, and RNase H enzymes with strand displacement activity [9,10]. The loop-mediated isothermal amplification (LAMP), recombinase polymerase amplification (RPA), helicase-dependent amplification (HDA), nucleic acid sequence-based amplification (NASBA), rolling circle amplification (RCA), and multiple strand displacement amplification (MDA) are examples of isothermal amplification methods [11,12,13,14,15,16]. Since the isothermal amplification method does not require a thermal cycler, it has high detection efficiency and enables rapid detection [17]. Additionally, the applicability of point-of-care testing (POCT) is also high, and it is effectively applied for detecting food-borne pathogens [12,17].

Although molecular-based diagnostics are the most efficient and effective methods for detecting food-borne pathogens, the detection efficiency is proposed to be decreased because of different inhibitors that produce false-negative results [18,19]. These inhibitors originate from various foods and environmental or clinical samples used in the analysis, and from some compounds used during the amplification reaction and extraction process, in addition to reaction conditions [19,20]. Also, fluorescent dye or DNA-binding dye used for amplicon detection after amplification can serve as inhibitors during the process of isothermal amplification [21,22].

PCR inhibitors from foods have been studied and reported upon [19,21,22]. In addition, the isothermal amplification inhibitors have continuously been reported upon. However, it is not enough to report on the inhibitory mechanism and removal strategy compared to PCR. Therefore, isothermal amplification techniques used in food-borne pathogen detection and different inhibitors that interfere with isothermal amplification are described.

## 2. Isothermal Amplification Methods

LAMP is conducted in the presence of *Bst* DNA polymerase, deoxyribonucleotide triphosphate (dNTP), and 4–6 specific primers (inner and outer primers) that recognize 6–8 specific regions [23]. The *Bst* DNA polymerase removes the need for high thermocycling because of its activity at 50–70 °C, and the DNA polymerase is less sensitive to food-origin inhibitors [24,25]. The use of two outer primers (forward outer primer; F3, and backward outer primer; B3), two inner primers (forward inner primer; FIP, and backward inner primer; BIP), with additional loop primers (forward loop primer; LF, and backward loop primer; LB) also allows sequence-specific detection, improves specificity, and accelerates assay specificity to amplification targets [26]. However, although many long primers increase the reaction yield, a risk of primer dimerization owing to nonspecific interactions simultaneously exists [9,27]. To prevent primer dimerization, adding dimethyl sulfoxide (DMSO) and betaine is used as a strategy to decrease nonspecific interactions, thereby giving stability to oligonucleotides [28]. The use of additional compounds can amplify nucleic acids without non-specific amplification, and exponential nucleic acid amplification is achieved while forming loops within 40–60 min at a constant temperature of 60–65 °C [29]. Then, the amplified product can be detected using turbidity measurement, gel electrophoresis, colorimetry, electrochemiluminescence, lateral flow assay (LFA), and real-time monitoring [30,31].

RPA is conducted while a recombinase protein UvsX from T4-like bacteriophages forms a complex with primers in the presence of ATP and a crowding agent, such as polyethylene glycol (PEG) [32]. The crowding agent prevents spontaneous recombinase-primer degradation, thereby allowing amplification to begin. It also enhances the amplification efficiency by improving catalytic activity of the enzyme [33,34]. Therefore, using long primers (up to 45 nucleotides) can form secondary structures and potential primer artifacts; recommended length of RPA primers is 30–35 bases [32,35]. RPA is conducted at relatively low and constant temperatures of 37–42 °C for 20–40 min, and detection of amplicons are performed through gel electrophoresis, flocculation assay detection, LFA, electrochemical assay, chemiluminescent assay, and silicon microring resonator (SMR)-based photonic assay, and real-time monitoring [12,34,36].

HDA reacts with helicase, two primers, and two accessory proteins; methyl-directed mismatch repair (MutL), and single-stranded DNA (ssDNA) binding (SSB) proteins that stimulate UvrD helicase activity above ten-fold [37,38]. During the HDA replication process, the helicase unwinds double-stranded DNA (dsDNA) for the denaturation. In this step, the accessory proteins are required to bind and stabilize the ssDNA for the prevention of recombination of the complementary strand, thereby allowing primer hybridization [10]. The HDA reaction is divided into two systems: the mesophilic form of HDA (mHDA) and the thermophilic form of HDA (tHDA). The mHDA reacts at a medium temperature of 37 °C using UvrD helicase/Exo-Klenow polymerase; the tHDA reacts at a higher temperature (60–65 °C) using a thermostable Tte-UvrD helicase/*Bst* DNA polymerase [39]. Compared to mHDA, tHDA has a higher sensitivity and efficiency, and simplifies the reaction because it does not need MutL and SSB proteins to stabilize the DNA sequence [11]. The magnesium ion, which serves as a cofactor for the helicase and polymerase, is also used in HDA to increase the enzyme activity, thereby making the enzymes compatible with structurally modified primers [9,40]. Subsequently, amplification products are detected using gel electrophoresis, colorimetry assay, LFA, and real-time monitoring [11].

NASBA targets 16s rRNA genes or mRNA transcripts for bacterial detection, enabling the analysis of bacterial viability [16,41]. NASBA amplifies single-stranded RNA using two primers and three enzymes (avian myeloblastosis virus reverse transcriptase (AMV-RT), RNase H, and T7 DNA-dependent RNA polymerase (DdRp)) [42]. While AVM-RT generates complementary complementary DNA by extending primers, dsDNA is formed through RNase H. However, T7 DdRP recognizes the exposed T7 promoter of the dsDNA and initiates transcription to initiate the reaction [42]. Since NASBA enzymes are heat labile, amplifications can be performed at a relatively low temperature, with optimal conditions of 41 °C for 1.5–2 h [43]. The low reaction temperature of NASBA, however, can produce to result in false-positive results because of nonspecific primer interactions. Nevertheless, adding DMSO and betaine can prevent this limitation [44,45]. Subsequently, amplification products are detected using gel electrophoresis, enzyme-linked immunosorbent assay (ELISA), enzyme-linked gel assay, electrochemiluminescent (ECL), and real-time monitoring with molecular beacons [9,46]. When the clustered regularly interspaced short palindromic repeats (CRISPR)/Cas system and NASBA are combined, RNA can be detected with high sensitivity through the NASBACC (NASBA-CRISPR Cleavage) system [47]. 

RCA has high specificity and sensitivity targeting RNA and DNA [48]. The RCA reaction is conducted using DNA/RNA polymerase (phi29 DNA polymerase or T7 RNA polymerase), short DNA or RNA linear single-stranded primer, circle template, and ligase [49]. DNA/RNA polymerase produces a long single-stranded RCA product (RCAP) complementary to a circular template. Unique oligonucleotide padlock probes (PLP) and T4 DNA ligase or special ssDNA ligase synthesize bacterial single- or double-stranded RNA/DNA templates into single-stranded circular DNA [9]. The various modified RCA systems have been developed for efficient amplification: a linear-RCA (LRCA) or an exponential-RCA (ERCA), such as multiply-primed RCA (MPRCA), hyperbranched RCA (HRCA), and primer-generation RCA (PG-RCA) [42]. The saltatory RCA (SRCA), a simplified form of RCA, has been developed, requiring ligase and PLP for cyclization [14,50]. RCA generally reacts for 1–1.5 h at 30–65 °C depending on the reaction system, and amplification products are detected using gel electrophoresis, colorimetry, and real-time monitoring [42].

MDA randomly and massively amplifies single-cell genomic DNA and is compatible with whole genome amplification (WGA) [51,52]. MDA is conducted using modified random hexamer primers, phi29 DNA polymerases (strand-displacing DNA polymerase from bacteriophage Ø29), denatured template DNA, and dNTPs [53]. The modified random hexamer primers eliminate the need to design target-specific primers are designed to anneal to random areas on each strand of the target DNA, thereby forming hyperbranched intermediates and dsDNA amplicons after exponential amplification [54]. The phi29 DNA polymerase enables amplification at a relatively low temperature (typically 30 °C) because of its high strand displacement activity; it has a higher replication fidelity and lower error rate than *Taq* DNA polymerases and *Bst* DNA polymerases [13,55]. Furthermore, the addition of PEG to the reaction for high-efficiency MDA causes molecular crowding, which enables sensitive allele detection in multiplex short tandem repeat genotyping [56].

**Figure 1:**
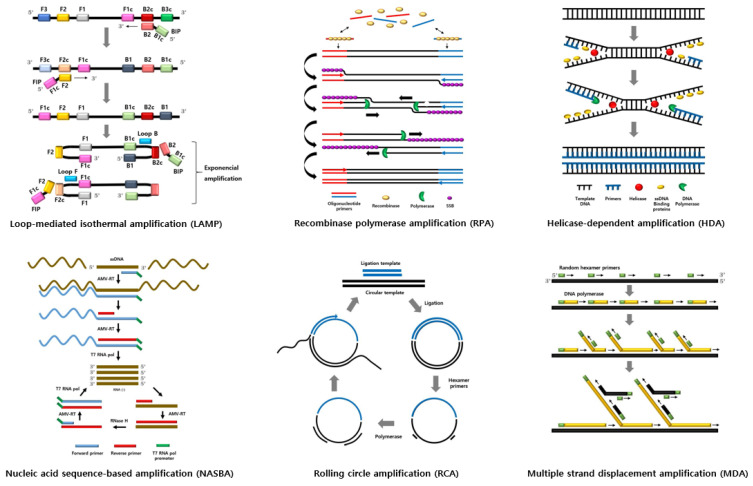
Schematic diagram of isothermal amplification techniques.

Schematic diagram and summary of isothermal amplification techniques are shown in Figure 1 and Table 1.

## 3. Inhibitors That Originate from Food during the Isothermal Amplification Process

Isothermal amplification methods, with a high detection efficiency, sensitivity, and specificity using geometrically amplifying DNA/RNA, have been widely used to detect food-borne pathogens (Table 2) [58]. However, complex food matrices can inhibit the detection of these food-borne pathogens. Therfore, food-origin inhibitors possess inhibitors that make the DNA/RNA extraction challenging, denature DNA or polymerases, or bind magnesium ions to interfere with amplification [22,24,25]. Furthermore, it has been reported that these inhibitors are complex polysaccharides, phenolic compounds, and calcium ions [19,26].

Polysaccharides in vegetables, fruits, and seafood can inhibit nucleic acid amplification [19]. Cationic polysaccharides can therefore interfere with DNA amplification initiation to inhibit DNA polymerase activity, thereby inducing competitive binding at the primer’s binding site owing to their binding properties to anionic particles, such as the template DNA of bacteria [27]. Interference is due to the electrostatic interaction of the anionic phosphate and carboxyl groups in the lipopolysaccharide (LPS) of the extracellular membrane with the cationic molecule, particularly in gram-negative bacterial cells [27,59]. Similar to this inhibition mechanism, chitosan, contained in crab and shrimp, inhibited LAMP by aggregating negatively charged template DNA molecules [27]. A study also reported that polysaccharides in rice interfered with LAMP by inhibiting activity of *Bst* DNA polymerase [60]. Additionally, polysaccharides can inhibit nucleic acid amplification by forming high-viscous DNA solutions through mechanical spatial entanglements with DNA [61]. This inhibition works because DNA can be denatured by viscous formation processes. High-viscous DNA solutions can inhibit subsequent DNA quantification and amplification [62]. In a previous study, the column/membrane of the DNA extraction kit was blocked because of the highly viscous DNA solution formed by soybean polysaccharides, thereby leading to low DNA yield [63]. Recently, commercial DNA extraction kits have increased the DNA extraction yield by suppressing the formation of viscous DNA [64,65]. However, since this method controls the high-viscosity of a DNA solution formed after the inhibition mechanism of polysaccharides, it is impossible to fundamentally remove the inhibition before forming the high-viscosity DNA solution that interferes with amplification. Therefore, future research should alleviate the inhibitory mechanism of polysaccharides in food in advance.

Phenolic compounds contain secondary metabolites, such as polyphenols, tannic acids, caffeic acids, and chlorogenic acids, found in strawberries, citrus fruits, and potatoes [19,101]. These secondary metabolites can react with primary reactive residues of enzymes/proteins during amplification to change their intrinsic properties [102]. Consequently, the extraction of high-purity DNA is complex, and amplification can be inhibited [19,26]. Tannins are readily oxidized to bind proteins and nucleic acids irreversibly, thereby forming high molecular weight complexes, which makes DNA extraction difficult [103,104]. It is possible to prevent oxidation of polyphenols by adding polyvinylpyrrolidone and sodium acetate, and by adding soluble PEG to prevent the formation of free radicals, high-purity DNA can be obtained [105,106]. However, besides this, phenolic compounds can inhibit amplification by interfering with the activity of DNA polymerases, DNA restriction enzymes, and RNA-dependent reverse transcriptases [19,57].

High concentrations of calcium ions in milk can inhibit nucleic acid amplification through competitive binding to DNA polymerases with magnesium ions, a cofactor of DNA polymerase and helicase [104]. Milk-based amplification inhibition occurred during MDA for detecting *S.* Typhimurium and *Cronobacter sakazakii* [107]. Additionally, MDA was amplified in the presence of 14–16% milk, which was even lower than the quantity which RPA was inhibited (15–25%), thereby confirming the weak resistance to food matrices. Additionally, Santiago–Felipe et al. [57] reported that this inhibition could be because of calcium ions in milk.

## 4. Inhibitors Originating from Isothermal Amplification Processes

Various inhibitors repress the isothermal amplification technique for detecting bacteria, including food-borne pathogens. These inhibitors originate from amplification or detection processes and the food metrix (Table 3).

*Bst* DNA polymerase and helicase that enables isothermal amplification require a relatively high concentration of magnesium ions of about 4–8 mM compared to *Taq* DNA polymerase [38]. However, magnesium ions can inhibit molecular amplification depending on the concentration in the reaction mixture [110]. Murakami et al. [110] reported that by increasing the background signal amplification in RCA, the concentration of magnesium ions inhibited the signal during target gene amplification. Nb.BsmI, a nicking enzyme, needs magnesium ions to enhance amplification efficiency. Therefore, optimizing magnesium ion concentrations (4–6 mM MgSO_4_) and decreasing dNTPs and DNA polymerase concentrations can decrease background signal amplifications. Doseeva et al. [109] conducted a study to alleviate magnesium ion-dependent inhibition because the concentration of magnesium ions affected the amplification efficiency of tHDA. Results indicated that as the concentration of magnesium ions and that of the dATPs improved, the signal-to-noise ratio increased 1.5–2.0 times. It was also observed that the optimal concentrations of MgSO_4_, dATP, and dNTP were 4, 3, and 0.4 mmol/L, respectively. Additionally, betaine, DMSO, and sorbitol, which help the combined effect of magnesium ions and dATP during molecular amplification, were added to increase the efficiency and specificity of amplification in tHDA.

The inappropriate concentration of primers can inhibit molecular amplification processes. Thus, SSB proteins either inhibit the strand exchange activity of recombinase T4 UvsX or compete with recombinase proteins in RPA [118]. Additionally, primers for one target can inhibit the amplification of another target [32]. Therefore, it is essential to optimize primer concentration [32,36].

DNA/RNA templates as amplification targets can also inhibit molecular amplification. RPA reactions are more sensitive to inhibitors when the DNA concentration of the reaction mixture for amplification is close to that of the detection limit or when the background DNA concentration is high [36]. Additionally, in the initial denaturation step for denaturing the secondary structure of mRNAs before NASBA amplification, short or partially digested DNA molecules can be denatured and inhibited, thereby acting as a substrate for NASBA [118]. Simpkins et al. [84] overcame the induction of mRNA degradation using RNase enzymes in cells through pasteurization and a 15 min incubation before the nucleic acid extraction. The combination of RNase A, treatment, and molecular labeling based real-time NASBA assay successfully detected viable *L. monocytogenes* cells in meat and salmon products without an initial denaturation step that can cause inhibition. Additionally, Sidoti et al. [112] suggested that adding 60% DMSO to NASBA primers and beacon mixtures, although not food samples, improved results through oligonucleotide stabilization maximization.

The colorimetric method, which is widely used to identify amplicons after amplification, can be inhibited using some compounds, such as intercalating dyes or cationic polymers [22]. SYBR Green I is a fluorescent dye widely used for colorimetric isothermal amplification detection because of its high detection sensitivity of amplified nucleic acids [15,114,119]. Therefore, it has been reported that if such a fluorescent dye was added directly to the reaction mixture before LAMP and reacted during amplification, the amplification process will be inhibited. However, if a fluorescent dye was added after amplification, the amplification result can vary owing to cross-contamination and aerosol contamination [114,120]. This inhibition was due to the strong bonding of the dye to ssDNA, and the ability to bind LAMP and loop primers in real-time to prevent primer annealing or interference with the activity of *Bst* DNA polymerase [121]. Hence, to prevent this inhibition, studies have allowed these fluorescent dyes to react after amplification in a closed tube system [114,122]. Wax capsules containing fluorescent dye have been reported too. They preserve the fluorescent dyes before amplification and allow the fluorescent dyes to react with amplicons when the wax melts after amplification [114]. Additionally, a study was also reported in which the fluorescent dye was applied to the inner part of the tube’s cap. Then the tube was rotated after amplification to allow the fluorescent dye to react with the amplicon [121]. Similarly, nonmutagenic noncytotoxic fluorescent intercalator (EvaGreen) had less amplification inhibition capacities compared to SYBR Green I because of its characteristic of fluorescing only when bound to dsDNA. Thus, it was suggested that it could replace SYBR Green I [11,123].

DNA-binding dyes can also inhibit detection after nucleic acid amplification [122]. When polyethyleneimine (PEI), a cationic polymer widely used as a nucleic acid precipitating agent for nucleic acid purification, is added to the amplicon to precipitate the insoluble LAMP amplicon-PEI complex, it enables the sequence-specific visual detection of trace quantities of nucleic acids [124]. However, since the insoluble DNA-PEI complex neutralizes the negative charge of DNAs and consequently inhibits LAMP, it should be added after amplification [115]. Metal ion indicators, such as MnCl_2_ and calcein, can also be added to the reaction mixture before amplification, thereby enabling amplification and detection to be conducted in a closed tube system. Wastling et al. [115] compared the sensitivity of LAMP using a metal ion indicator and calcein supplemented with MnCl_2_. Results indicated a lower LAMP sensitivity than calcein alone.

During electrochemical monitoring developed to digitize amplicon detection, amplification inhibition is proposed to occur due to partial defects in redox active compounds [22]. Methylene blue (MB) has a lower LAMP amplification efficiency than other redox molecules with binding activity (10^4^–10^5^ M^−1^) to dsDNA, a Hoechst 33258 redox molecule, which is unsuitable for real-time monitoring of LAMP, as it limits DNA amplification and detection in the solution phase, and strongly inhibits enzyme activity of polymerase for endpoint DNA detection [116]. Replacing DNA dyes can improve this LAMP inhibition. Osmium redox showed high binding activity and stability during isothermal amplification. In contrast, real-time electrochemical detection without inhibition detected ruthenium hexamine (RuHex) because it lacked an intercalating ligand and bound electrostatically to the anionic dsDNA backbone [125,126]. Ahmed et al. [127] used RuHex to successfully detect without inhibition because it can be used as an indicator to monitor LAMP amplicons instead of immobilizing the probe to the electrode’s surface. Additionally, the voltammetry mode, developed based on the electrochemical reaction of free dGTP molecules in the carbon nanotube array electrode, can be used without inhibition because no secondary indicator exists. Polydopamine-doped paper disks eliminated LAMP inhibitors by reacting with milk components and calcium ions, thereby enabling the simultaneous multiplex detection of food-borne pathogens without inhibition [43,117,120]. Alternatively, the electrochemical monitoring of HDA uses an electroactive intercalator instead of a fluorescent probe. Here the redox probe inhibits the amplification of the specific sequence of the *E. coli* plasmid, so that the amplification rate of electrochemical monitoring would be slower than that of fluorescent real-time amplification [11].

## 5. Conclusions

Inhibitors originating from food matrices, DNA extraction reactions and nucleic acid amplification reactions can adversely affect isothermal amplification techniques by inhibiting amplification to detect food-borne pathogens. Nevertheless, due to the complex nature of the food matrix, food-origin inhibitors, including inhibition mechanisms for molecular analysis, have not been fully characterized yet. Additionally, studies on the inhibitory mechanisms of inhibitors obtained during isothermal amplification processes are still insufficient and have not been clearly established. The above-mentioned removal strategies for inhibiting nucleic acid amplification are rather limited and uneconomical because they only target specific inhibitors, and have not been proven to be applicable to various inhibitors. Therefore, further studies on nucleic acid amplification inhibitors and inhibitor removal strategies are needed to detect food-borne pathogens in food using isothermal amplification technologies without inhibition of nucleic acid amplification inhibitors. In the future, these studies will lead to the manufacture of a ready-to-use kit that simultaneously purifies and removes inhibitors. In this case, efficiency is guaranteed for accurate detection of food-borne pathogens in complex food matrices and for the POCT application of these isothermal amplification technologies.

## Figures and Tables

**Table 1:** Summary of comparison among various isothermal amplification methods.

Isothermal Amplification Methods	Number of Primers	Number of Enzymes	Pre-Heating	Working Temperature (°C)	Reaction Time (min)	Target Template	Amplicon	Resistance to Inhibitor	Reference
LAMP	4–6	1	No	60–65	40–60	DNA	DNA	High	[29]
RPA	2	2	No	37–42	20–40	DNA	DNA	Low	[12]
HDA	2	1 (mHDA), 3 (tHDA)	No	37 (mHDA), 60–65 (tHDA)	100–120	DNA	DNA	High	[11]
NASBA	2	2–3	Yes	41	90–120	RNA	RNA, DNA	Low	[43]
RCA	1	1	Yes	30–65	60–90	Circular DNA	DNA	Low	[42]
MDA	Random hexamer primers	1	No	35	270	Circular or linear DNAs	Ramified double–stranded DNAs	High	[57]

Abbreviations used in the table: LAMP, loop-mediated isothermal amplification; RPA, recombinase polymerase amplification; HDA, helicase-dependent amplification; mHDA, mesophilic form of HDA; tHDA, thermophilic form of HDA; NASBA, nucleic acid sequence-based amplification; RCA, rolling circle amplification; MDA, multiple strand displacement amplification.

**Table 2:** Isothermal amplification processes used for detecting food-borne pathogens in foods.

Type of Food	Target Bacterial	Isothermal Amplification Method		Reference
		Nucleic Acid Amplification	Detection Method	
Meat	*Salmonella* spp.	LAMP	Intercalating dye	[5,66,67,68]
			Real-time	[69,70]
			LFA	[24,71]
		RPA	Real-time	[72,73,74]
		HDA	LFA	[37]
		NASBA	ECL	[75]
			Real-time	[16]
	*Escherichia coli* O157:H7	LAMP	Real-time	[76]
			LFA	[77]
		RPA	DNA-binding dye	[78]
	*Listeria monocytogenes*	LAMP	Intercalating dye	[79]
		RPA	LFA	[29,80,81,82]
		NASBA	ELISA	[83]
			Real-time	[84]
		MDA	LFA	[54]
	*Vibrio parahaemolyticus*	LAMP	LFA	[85]
	*Staphylococcus aureus*	LAMP	Intercalating dye	[67]
		HDA	Fluorescence	[86]
Seafood	*Salmonella* spp.	RPA	LFA	[87,88]
		NASBA	ECL	[75]
	*Escherichia coli* O157:H7	RPA	LFA	[29]
	*Listeria monocytogenes*	RPA	Real-time	[89]
			LFA	[29,80,82]
		NASBA	ELISA	[75]
			Real-time	[84]
	*Vibrio parahaemolyticus*	LAMP	Intercalating dye	[67,90]
			Real-time	[91]
		RPA	Real-time	[92]
			LFA	[29,88,93]
		MDA	LFA	[94]
	*Staphylococcus aureus*	RPA	LFA	[88]
Vegetable	*Salmonella* spp.	LAMP	Real-time	[70]
		RPA	Real-time	[74]
Dairy produce	*Salmonella* spp.	LAMP	Intercalating dye	[68,95]
			LFA	[71]
		RPA	Real-time	[96]
			LFA	[29]
			CRISPR/Cas12a	[97]
		HDA	LFA	[37]
		NASBA	ECL	[75]
			Real-time	[16]
	*Escherichia coli* O157:H7	LAMP	Intercalating dye	[95]
		RPA	Real-time	[96]
			LFA	[29,98]
	*Listeria monocytogenes*	RPA	Real-time	[99]
			LFA	[29,80,81,82]
		NASBA	ELISA	[83]
	*Vibrio parahaemolyticus*	LAMP	Intercalating dye	[95]
		RPA	Real-time	[96]
			LFA	[29]
	*Staphylococcus aureus*	LAMP	Intercalating dye	[95]
		HDA	Fluorescence	[86]
		NASBA	Real-time	[100]

Abbreviations used in the table: LAMP, loop-mediated isothermal amplification; RPA, recombinase polymerase amplification; HDA, helicase-dependent amplification; NASBA, nucleic acid sequence-based amplification; MDA, multiple strand displacement amplification; LFA, lateral flow assay; ECL, electrochemiluminescent; ELISA, enzyme-linked immunosorbent assay; CRISPR/Cas12a, clustered regularly interspaced short palindrome repeats/Cas12a.

**Table 3:** Inhibitors from the isothermal amplification reaction process.

Reaction Process	Inhibitors		Alleviation Strategies for Inhibition	Amplification Methods	Reference
Sample preparation and DNA extraction	Residual food metrix		Use the nucleic acid sample after dilution	NASBA	[75]
	CTAB used as extraction buffer		Use direct PCR buffers	RPA	[96]
Nucleic acid amplification	Concentration	Magnesium ions	Increase the concentration of magnesium ions	tHDA	[108,109]
			Add betaine, DMSO, and sorbitol to the reaction mixture		[37]
			Use 4–6 mM MgSO_4_, which is the optimal concentration for magnesium ions	RCA	[110]
		Primer	Optimize concentration of primer	RPA	[32]
				Multi-RPA	[111]
		Template or background DNA	Treat RNase A with pasteurization and 15 min incubation process before nucleic acid extraction	Real-time NASBA	[112]
			Add the primer stability enhancer to the primer and beacon mixture	NASBA	[113]
	Temperature	Temperature fluctuations	Optimize reaction temperature	MDA	[107]
		Heat denaturation	Substitute alkaline denaturation		[114]
Detection method	Colorimetric detection	SYBR Green I	Add fluorescent dyes after amplification	LAMP	[97]
			Use wax capsules containing the dye, which react after amplification		[114]
		PEI	Add PEI after amplification	LAMP	[115]
		Calcein	-		-
	Electrochemical detection	Redox active compounds (e.g., MB and Hoechst 33258)	Use other redox molecules (e.g., osmium redox and RuHex)	LAMP	[116]
			Use voltammeric mode		[97]
			Use polydopamine-doped paper disks		[117]

Abbreviations used in the table: NASBA, nucleic acid sequence-based amplification; CTAB, cetyltrimethyl ammonium bromide; PCR, polymerase chain reaction; RPA, recombinase polymerase amplification; DMSO, dimethyl sulfoxide; tHDA, thermophilic form of HDA; RCA, rolling circle amplification; MDA, multiple strand displacement amplification; LAMP, loop-mediated isothermal amplification; PEI, polyethyleneimine; MB, methylene blue; RuHex, ruthenium hexamine.

## Data Availability

Not applicable.
